# Congenital tremor and splay leg in piglets – insights into the virome, local cytokine response, and histology

**DOI:** 10.1186/s12917-022-03443-w

**Published:** 2022-09-16

**Authors:** Hedvig Stenberg, Stina Hellman, Lisa Lindström, Magdalena Jacobson, Caroline Fossum, Juliette Hayer, Maja Malmberg

**Affiliations:** 1 Department of Biomedical Sciences and Veterinary Public Health, Swedish University of Agricultural Sciences, SLU, P.O. Box 7028, 750 07 Uppsala, Sweden; 2 Department of Clinical Sciences, Swedish University of Agricultural Sciences, Box 7054, 75007 Uppsala, Sweden; 3 MIVEGEC, University of Montpellier, IRD, CNRS, Montpellier, France; 4 Department of Animal Breeding and Genetics, Swedish University of Agricultural Sciences, Box 7023, 750 07 Uppsala, Sweden

**Keywords:** Congenital tremor, Type A-II, Atypical porcine pestivirus, Splay legs, Sweden, Pigs, Piglets, Pathology, Immunology, Virome, IFN-α, STING

## Abstract

**Background:**

Atypical porcine pestivirus (APPV) is a neurotropic virus associated with congenital tremor type A-II. A few experimental studies also indicate an association between APPV and splay leg. The overarching aim of the present study was to provide insights into the virome, local cytokine response, and histology of the CNS in piglets with signs of congenital tremor or splay leg.

**Results:**

Characterization of the cytokine profile and virome of the brain in piglets with signs of congenital tremor revealed an APPV-associated upregulation of Stimulator of interferon genes (STING). The upregulation of STING was associated with an increased expression of the gene encoding IFN-α but no differential expression was recorded for the genes encoding CXCL8, IFN-β, IFN-γ, IL-1β, IL-6, or IL-10. No viral agents or cytokine upregulation could be detected in the spinal cord of piglets with signs of splay leg or in the brain of piglets without an APPV-infection. The histopathological examination showed no lesions in the CNS that could be attributed to the APPV-infection, as no difference between sick and healthy piglets could be seen.

**Conclusion:**

The results from this study provide evidence of an APPV-induced antiviral cytokine response but found no lesions related to the infection nor any support for a common causative agent.

**Supplementary Information:**

The online version contains supplementary material available at 10.1186/s12917-022-03443-w.

## Background

Atypical porcine pestivirus (APPV) is a newly detected neurotropic virus [1] that has been associated with congenital tremor type A-II through both experimental and natural infections [2–5]. The virus has also been associated with splay leg by experimental infections (Arruda et al., 2016, de Groof et al., 2016). However, APPV has never been demonstrated in naturally occurring cases of splay leg [6].

Both congenital tremor and splay leg are congenital disorders and their clinical signs are present directly from birth. Piglets born with congenital tremor present a varying degree of action tremor and, occasionally, ataxia. The lethality of congenital tremor ranges from around 10% to 30% at affected farms, mainly due to malnutrition or crushing by the sow [2, 3, 7]. The condition is reversible and piglets that survive until weaning often recovers completely [5, 8]. Based on the presumed aetiology and the pathoanatomical lesions, five known A-types of congenital tremor, characterized by hypomyelination and vacuolization in the white matter of the brain and one B-type associated with no microscopical lesions and no known causative agent are described [9–12].

Splay leg, spraddle leg syndrome, or porcine splay leg syndrome (PCS) is characterized by an impaired adduction of the hind limbs, and sometimes also the front legs [13]. The at-farm prevalence of splay leg ranges from 1–8% globally [14, 15], with a lethality up to 50% due to hypoglycaemia or mother overlying, crushing [16]. The impairment of the limbs is attributed to a hypomyelination of the spinal cord and the nerves innervating the affected muscles and a myofibrillar deficiency described as myofibrillar hypoplasia, an impaired muscular differentiation [13, 17, 18]. These histological findings are not pathognomonic for splay legs since they are subtle and can be seen in healthy neonatal piglets [16, 19]. Several risk factors, both extrinsic and intrinsic, are associated with splay leg *i.e.* hereditary factors [20], insufficient intrauterine nutrition [21, 22], induction of parturition [23], genetics [24, 25], and viral infections [2, 3, 26].

The virus that causes congenital tremor type A-II, APPV, belongs to the species *Pestivirus K* of the genus *Pestivirus* and the family *Flaviviridae* [1, 27]. It is a single-stranded RNA virus with a polyprotein consisting of 12 proteins: C (capsid protein), E^rns^, E1, E2 (envelope proteins), and non-structural proteins N^pro^, p7, NS2, NS3, NS4A, NS4B, NS5A, and NS5B [1, 27, 28]. The virus is known to induce a humoral immune response in affected pigs which is characterized by antibodies against the E2 and E^rns^ -proteins [29, 30] but, to date, nothing is known about the local inflammatory response in the CNS.

Although congenital tremor and splay leg are syndromes that have been known for decades and have a global distribution [5, 13, 15, 31, 32] knowledge of the pathology and causative agent(s) of these syndromes is still scarce. The aim of the present study was therefore to provide a more comprehensive description of natural cases of congenital tremor type A-II and splay leg, respectively, by high-throughput sequencing to characterize the virome of the brain tissue in piglets with signs of congenital tremor and the spinal cord tissue in piglets with signs of splay leg. In addition, histological examinations of the CNS were performed together with screenings for local cytokine production in the brain tissue of piglets suffering from congenital tremor and in the spinal cord tissue of piglets suffering from splay leg.

## Results

### Sequencing

Comparison of the results generated from Kraken 2 run against the non-redundant protein database (*nr*) to the DIAMOND run against the non-redundant protein database (*nr*) revealed in general similar results. However, the Kraken 2 run against the nucleotide non-redundant (*nt*) NCBI database was the only run that generated APPV hits in the piglets where the virus previously was demonstrated by PCR (Stenberg et al., 2020a). Hence, the Kraken 2 runs against the nucleotide non-redundant (*nt*) NCBI database were deemed to produce the most reliable results for this dataset and were therefore chosen for the taxonomic classification. An additional file shows the specific details for each sample (see additional file 1).

### Piglets with signs of congenital tremor (NovaSeq)

Metagenomic sequencing was performed on brain tissue originating from six piglets with clinical signs of congenital tremor. The sequencing was performed on the Illumina NovaSeq platform. Four piglets, PCR-positive for APPV-genome in the brain, and two piglets PCR-negative for APPV-genome [6] were selected.

After quality control and filtering, the sequencing generated between 5.71 and 72.67 million reads. Of these reads, on average 78% mapped against the *Sus scrofa* genome and were removed. After removal of the host genome, about 90% of the remaining reads could be taxonomically classified. A majority of the reads, approximately 80%, were of chordate origin, mainly *Sus scrofa*. About 6% of the reads were of microbial origin. Less than 1% of the reads were classified as viral. Specific alignment against the APPV-genome identified APPV-sequences in all the four PCR-positive samples but not in the two PCR-negative samples.

In the APPV-PCR positive piglets, the most abundant viral reads were reads classified as APPV. The sample with the highest number of viral reads had 140 reads classified as APPV or Pestivirus K. The reads classified by Kraken as APPV were extracted and blasted using BLASTn and generated only APPV hits. Considerably fewer reads classified as other viruses were detected. The second most common viral taxon detected was also identified in all samples. These were reads classified by Kraken as Human respovirus 1 with 16 reads, at the most, in one sample. The reads were blasted against the non-redundant database *nt*, using BLASTn and generated Human respovirus hits. Kraken also detected a few reads from one sample classified as HIV, but after blast validation, these turned out to be false positives.

In the APPV-PCR negative piglets, the total number of viral reads was lower than in the APPV-PCR positive piglets. Only one virus with more than 10 reads was detected; 17 reads of Human immunodeficiency virus 1 in one sample, were confirmed as false positives with BLAST.

At the contig level, more than 88% of the de novo assembled contigs in all samples were classified. The majority of the classified contigs were of chordate origin and less than 1% of the contigs were viral. The only virus with more than 1 contig was APPV which rendered, at the most, 10 contigs in one sample. The contigs classified as APPV by Kraken were extracted and blasted with web BLASTn and generated APPV hits. No viral contigs were detected in the APPV-negative piglets.

### Piglets with signs of splay leg (MiSeq and NovaSeq)

Metagenomic sequencing was performed on tissue originating from the thoracic part of the spinal cord from seven piglets with signs of splay leg; tissue from six of the piglets was subjected to sequencing on an Illumina MiSeq platform and tissue from one piglet was subjected to sequencing on an Illumina NovaSeq platform. All of these seven piglets were PCR-negative for APPV-genome in the spinal cord [6].

The MiSeq sequencing generated between 0.94 and 78.10 million reads per sample after quality control and filtering. Of these reads, about 80% of the reads could be mapped against the host genome and were removed. Of the remaining reads, approximately 80% could be taxonomically classified. About 35% of the reads were of chordate origin, mainly *Sus scrofa*. The majority of the classified reads, approximately 45%, were of microbial origin but less than 0.1% were classified as viral. Alignment against the APPV-genome did not generate any hits.

In this dataset, there was no detection of true viral reads. There were reads classified as viral by Kraken, but once the reads were extracted and submitted for a BLAST search only reads classified as Equine infectious anemia virus generated hits in BLAST. Virus classified as Equine infectious anemia virus is assumed to be a reagent contaminant commonly detected in metagenomic datasets [33]. The other reads classified as viral reads by Kraken did not generate viral hits when blasted but mainly hits from the pig genome.

At the contig level, more than 90% of the de novo assembled contigs were classified. The majority of the contigs were of chordate origin and less than 0.1% of the contigs were of viral origin. Viral contigs were detected in five of the six samples, however in very low numbers. The most abundant viral finding were contigs classified as Edafosvirus sp. and generated three contigs in one sample. When these contigs were blasted they aligned to the pig genome. One contig classified as Equine infectious anaemia virus was detected in all samples, when these contigs were submitted to BLAST they aligned to the reagent contaminant Equine infectious anaemia virus genome.

After filtering and quality control, the NovaSeq sequencing generated 2.07 millionreads. Approximately 79% of the reads could be mapped against the *Sus scrofa* genome and were removed. After host mapping was performed, 99.6% of the reads could be taxonomically classified. Of these were 44% of chordate origin and 53% were classified as microbial. Less than 0.01% of the reads were of viral origin. Reads from two viruses were classified by Kraken: Orthohepevirus A (Hepatitis E) and Influenza A virus but when blasted these reads did not generate viral hits. No viral contigs were generated from this dataset. Specific alignment against the APPV-genome did not generate any hits.

### Healthy piglets (MiSeq)

Metagenomic sequencing was performed on brain tissue originating from eight clinically healthy piglets, PCR-negative for APPV-genome, on an Illumina MiSeq platform.

For seven of the eight samples, the sequencing generated between 8.13 and 50.64 million reads per sample after quality control and filtering. One sample (sample 18) was underrepresented during sequencing and generated only 758.77 thousand reads. In all samples approximately 88% of the reads mapped against the *Sus scrofa* genome and were removed. Of the remaining reads, about 60% could be taxonomically classified. A majority of the reads were of microbial origin, most of them classified as bacterial. Less than 0.01% of the reads were classified as viral. Specific alignment against the APPV-genome did not generate any hits.

In all samples, a few reads were classified as viral by Kraken but after blast validation, all of them turned out to be from the *Sus scrofa* genome. This could stem from misclassification, due to how Kraken’s algorithm works, or from the fact that some sequences in the databases are wrongly annotated.

At the contig level, more than 30% of the de novo assembled contigs were classified. A majority of the contigs were of microbial origin and less the 1% of the contigs were classified as viral. A few viral contigs were detected in all samples, but after blast validation almost all of them turned out to be from the *Sus scrofa* genome. One contig classified as Equine infectious anemia virus was detected in all samples and could be blast validated as Equine infectious anemia viru and thus most likely a reagent contaminant [33]

### Pathology

#### Gross findings

The necropsy of the healthy piglets, the piglets with signs of congenital tremor, and the piglets with signs of splay leg revealed no gross lesions. The results of the necropsy are described in detail by Stenberg et al. (2020a).

#### Histopathologic evaluation of brain and spinal cord

**Fig. 1:**
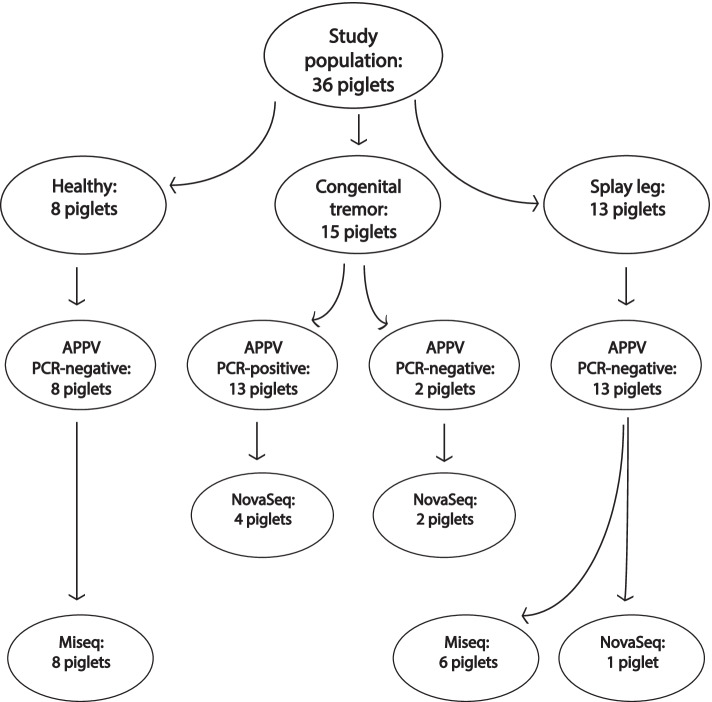
Flow chart showing the number of sequenced samples. A total number of 21 piglets were subjected to sequencing; eight healthy, six with signs of congenital tremor, and seven piglets with signs of splay leg. The piglets were selected to represent all sampled farms and litters

Scattered within the white matter at all levels of spinal cord and brain were a few multifocal, small vacuoles with variable shape and size. The vacuoles were randomly distributed with no associated cell reaction. In the brain, vacuoles were most common in cerebellum. In the spinal cord, vacuoles were more commonly seen, as compared to the brain. Vacuoles were present in equal numbers in the brain and spinal cord in healthy piglets and in piglets with signs of congenital tremor or splay legs. (Fig. 1, A & B).

The cerebellum and spinal cord from three piglets with high virus load and from one control piglet, were further investigated for myelin loss and changes and/or loss of nissl substances using luxol fast blue stain and cresyl violet stain. There were no pathologic findings consistent with a decreased amount of myelin or loss of nissl substance within the neurons, the staining intensity of the tissue being similar in the sick and healthy piglets. (Fig. 1, C & D).

### Transmission electron microscopy

Tissue from the cerebellum from two piglets (one with signs of congenital tremor and one clinically unaffected) were subjected to transmission electron microscopic examination.

**Fig. 2:**
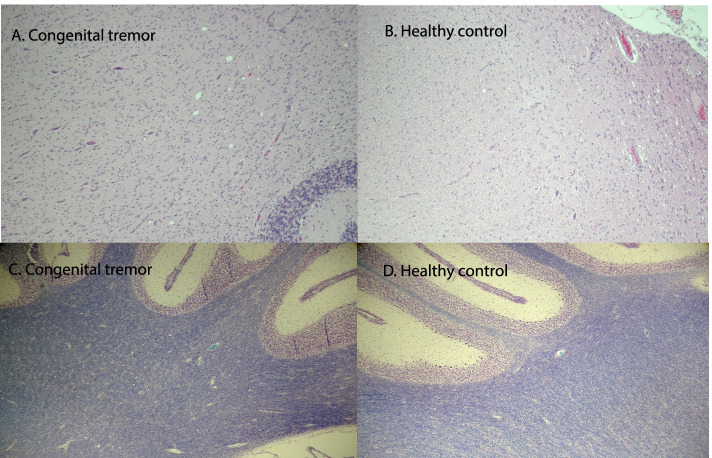
Cerebellar tissue from a piglet with signs of congenital tremor and a healthy control piglet. A and B: HE-stained cerebellar tissue from a piglet with signs of congenital tremor and a healthy control piglet. A few multifocal, small vacuoles with variable shapes and sizes can be seen in equal numbers in the sick and healthy piglet. Scale bar: 200 µm. C and D: Cerebellar tissue from a piglet with signs of congenital tremor and a healthy control piglet stained with luxol fast blue stain and cresyl violet stain. No myelin loss or loss of nissl substances can be seen. Scale bar: 500 µm

The piglet with signs of congenital tremor showed degenerated mitochondria, a mild separation of myelin lamellae, and small sporadic vacuoles in the neuropil. The degenerated mitochondria, characterized by swelling and loss of crista structure, were mainly present in the oligodendrocytes but also occasionally seen in the axons (Fig. 2).

In the cerebellar tissue of the clinically unaffected piglet, minor ultrastructural changes such as mild separation of myelin lamellae were detected.

### Cytokine analyses

The expression of cytokine genes associated to viral infections was studied in the brain tissue from piglets with signs of congenital tremor, positive for APPV (*n* = 13) and compared to the cytokine gene expression in samples from five of the clinically healthy piglets. For comparison, the cytokine genes that were found to be upregulated in these APPV-infected piglets were also analysed in spinal cord tissue from piglets with signs of splay leg (*n* = 12) and in the brain tissue from the two APPV-negative piglets displaying clinical signs of congenital tremor.

### Cytokine profile of APPV-infected brains

**Fig. 3:**
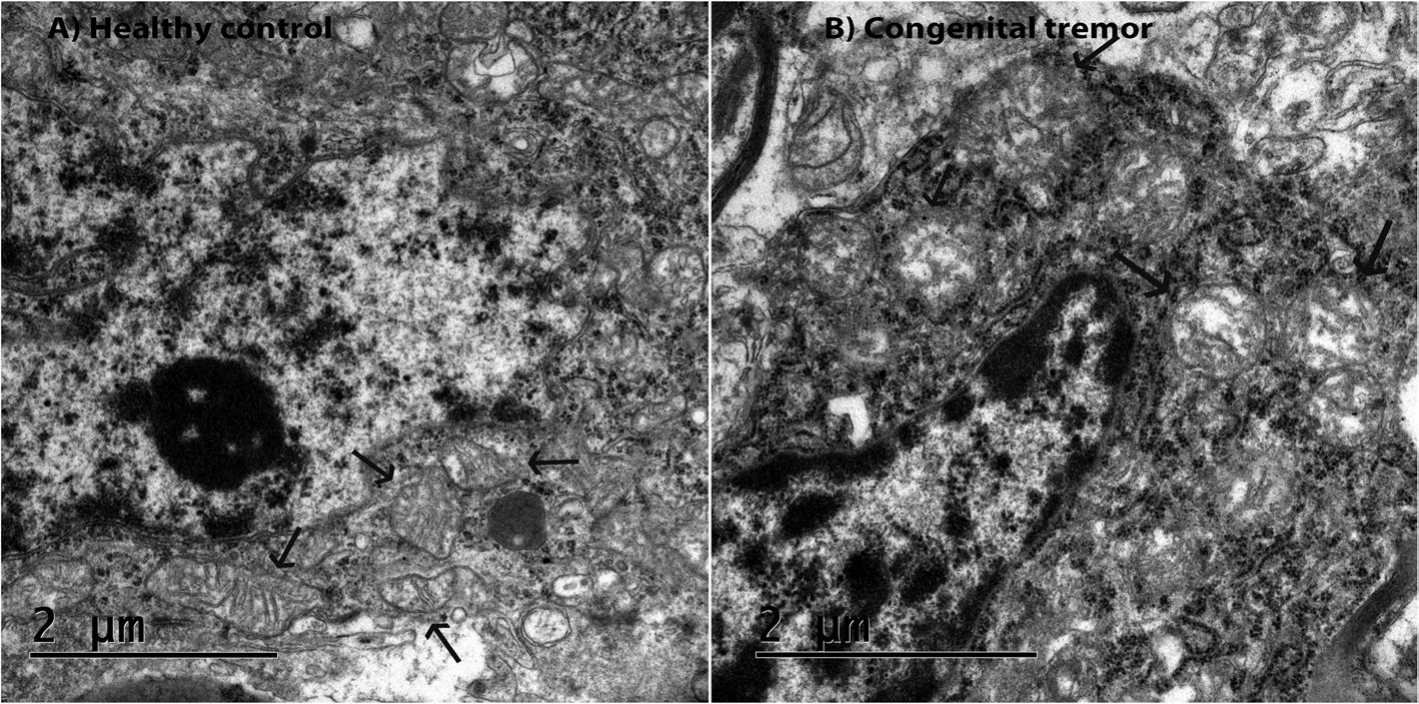
Oligodendrocytes from cerebellar tissue visualised by transmission electron microscopy. Transmission electron microscopic examination of cerebellar tissue from a piglet with signs of congenital tremor and a healthy control piglet. Mitochondria are marked with black arrows. In the healthy piglet, mitochondria are normal in size with a prominent crista. Numerous degenerated mitochondria, swollen with loss of crista, can be seen in the oligodendrocyte of the sick piglet

The expression of selected cytokine genes (CXCL8, IFN−α, IFN-β, IFN-γ, IL-1β, IL-6, IL-10, and STING) in brain tissue of APPV-positive pigs with signs of congenital tremor was related to the gene expression in brain tissue from five of the healthy piglets. Of these, the gene encoding STING was significantly up-regulated compared to the healthy control group. An up-regulation of IFN-α was also indicated but not statistically significant. (Fig. 3). However, a bivariate analysis found a significant correlation between the expression of STING and IFN-α (r = 0.824, *p* < 0.0001). The gene encoding IFITM3 was detected in all brain samples, but not differentially expressed (FC: 1.1 ± 1.9) compared to the healthy control group (FC: 1.4 ± 1.5). Expression of the genes encoding CXCL8, IFN-β, IFN-γ, IL-1β, IL-6, and IL-10 were not detected in the brain tissue of APPV-positive piglets despite that these genes were detected, although at high Cq values, in the brain tissue of the healthy piglets.

Significant upregulation, *p* < 0.05, of STING encoding gene in piglets PCR-positive for APPV and with signs of congenital tremor compared to the healthy control piglets. An up-regulation of IFN-α is indicated, however not statistically significant.

For the spinal cord tissue from piglets with signs of splay leg, the three genes that were detected in the APPV-positive piglets; STING, IFN-α, and IFITM3 were analysed but no expression of these genes were found. Thus, the presence of APPV appears to induce a STING-associated cytokine profile in porcine brain tissue. Supporting this, STING and IFN-α were not differentially expressed in the brains of the two CT piglets negative for APPV.

## Discussion

The present study aimed to characterize the virome, histopathology and cytokine profile of the CNS in piglets with signs of congenital tremor or splay legs. Comparison of these data from piglets with the two diseases indicated that congenital tremor and splay legs have different pathogenesis and, likely, different causative agents, despite sharing some clinical traits. In the present study, APPV was only detected in the brain tissue of piglets with signs of congenital tremor, and no virus or cytokine upregulation was detected in the spinal cord from piglets with splay legs, indicating that APPV is not the causative agent for this syndrome in natural infections.

Given that most piglets with congenital tremor type A-II recover fully from their signs and clear the APPV-infection [29] there has to be a functional immune mechanism restricting the viral replication. In accordance, transcriptional analysis of brain tissue from piglets with signs of congenital tremor and a concurrent APPV-infection revealed an upregulation of the gene encoding STING, a transmembrane protein at the endoplasmic reticulum involved in the activation of type-I interferons [34]. Interestingly, upregulation of STING could be correlated with an increased expression of the gene encoding IFN-α, indicating that APPV elicits antiviral innate immune reactions in the piglet brain. This antiviral response can be directly associated with APPV, as this was the only viral finding in the metagenomic sequencing of the brain tissue. The concept of the APPV-associated cytokine profile is further supported by the lack of upregulation of these cytokines in piglets where APPV was not found.

Interestingly, the upregulation of STING in the APPV infected brain is similar to the immune response in the brain following infection with the Zika virus, which is, similar to APPV, a neurotropic, small, enveloped ssRNA + virus [35, 36]. Zika infection is known to induce upregulation of STING [37], activate anti-viral autophagy [37], and stimulate the local immune response to increase the production of type-I interferons, such as IFN-α, in the brain. Although STING mainly has been associated with the recognition of cytosolic DNA [34], these mechanisms make STING important to the restriction of both RNA and DNA virus replication [38–40].

Stimulation of type-I IFN genes through STING has to be carefully regulated since an excessive production or continuous occurrence of type-I IFNs, *e.g.,* IFN-α, not only generates an anti-viral state in the cells of the surrounding tissue but may trigger tissue damage, especially in the brain [41]. A recent study on the immune response towards herpes simplex virus type-1-infection in the CNS suggests that the cGAS/STING pathway in the brain has a regulatory effect to protect the tissue from damage due to high IFN type-I activity [42]. This regulatory “feed-back loop” will make type-I IFN producing immune cells, microglia, in particular, undergo STING-dependent apoptosis if the immune-stimulatory signals are too intense *e.g.,* if the viral load is high [42]. Such a protective mechanism may be one possible explanation for the lack of extensive histopathologic lesions such as hypomyelination, apoptosis, or nerve cell damage in the CNS of piglets with signs of congenital tremor, despite a high presence of APPV in the tissue. However, if this is due to STING-mediated regulation remains to be elucidated.

Commonly, congenital tremor type A-II has been associated with histopathological lesions such as vacuolization of the white matter and hypomyelination in the brain [4, 10, 43–45]. These types of lesions were, however, not seen in the brain or spinal cord of any of the Swedish piglets. Interestingly, the histopathological lesions previously described by others are similar to descriptions of artifacts due to delayed fixation of the tissue. These artifact vacuoles and myelin alterations are often particularly prominent within the white matter of the CNS [46, 47]. In addition, it should be noted that the studies where severe lesions have been recorded lacks descriptions of the time from death to removal and fixation of the CNS tissue. Further, in these studies, the occurrence of non-APPV viral co-infections cannot be excluded. Reports of these lesions, therefore, need to be interpreted with caution. In the present study, measures were taken to avoid bias related to post-mortem autolysis. The pigs were brought alive to the Department of Pathology and euthanized immediately before necropsy and fixation of tissue. Thus, it can be speculated that the severity of the APPV-associated lesions in the CNS might not be as extensive as previously described. Nevertheless, the TEM examination in the present study revealed degenerated mitochondria, mainly in the myelin-producing oligodendrocytes, as well as mild myelin disruption in the cerebellum. This is indicative of a pathological process involving the myelin that might induce CNS-derived signs such as shaking.

The APPV associated upregulation of STING in the present study is further supported by the metagenomic sequencing results that ensure the absence of non-APPV viral co-infections. Overall, the metagenomic sequencing resulted in a low number of viral reads, both in the brain and spinal cord. The most obvious finding that emerged from the metagenomic analysis is that APPV could be detected in the brain tissue of APPV PCR-positive piglets with signs of congenital tremor. In these NovaSeq sequenced samples, reads classified as human respovirus-1 were also detected. Human respovirus causes a seasonal upper respiratory tract infection, common in humans [48] and the finding is probably caused by human contamination during library or sequencing preparation. Another probable contaminant was detected in the Miseq generated datasets, *i.e.,* in the datasets of the healthy piglets and the piglets with signs of splay leg; reads and contigs classified as Equine infectious anemia. The common discovery of reads and contigs classified as Equine infectious anemia virus in datasets produced by metagenomic next-generation sequencing has recently been attributed to a reagent contaminant, a novel reagent-associated lenti-like virus [33]. Phylogenetic analysis of this novel reagent-associated lenti-like virus shows that it is closely related to and clusters with several known sequences of Equine infectious anemia virus, making it a recurrent classification and alignment error in datasets [33].

No true viral hits could be detected in the APPV-PCR negative piglets with signs of congenital tremor, the piglets with signs of splay leg, or the healthy piglets. It can therefore be assumed that the sequencing depth was enough to detect a clinical infection as well as obvious contamination. However, it may be speculated that part of the full CNS-virome, such as phages or endogenous retroviruses, was lost during pre-preparation and library preparation. Almost all pigs carry porcine endogenous retroviruses incorporated in their chromosomes [49, 50] but these viruses will not be detected in datasets if precautions are taken to remove the host genome *i.e.,* pig genome, as done in the present study. To date, there are no published studies on the porcine CNS-virome available for comparisons with the results from the sequencing in the present study, since publications describing the porcine virome, with a few exceptions, mainly focus on organs relevant for xenotransplantation [49].

The outcome of the metagenomic and transcriptional analyses in the present study provides no evidence for a common causative agent or pathogenesis of splay leg and congenital tremor, although the diseases may have some common clinical traits. This result is contrary to previous studies *e.g.*, by Arruda et al. (2016) or de Groof et al. (2016), who suggested that congenital tremor and splay leg both can be induced by a transplacental infection with APPV. This discrepancy may be attributed to the fact that Arruda et al. (2016) and de Groof et al. (2016) conducted experimental infections, either by inoculation of APPV directly to the foetal amniotic vesicle or infection of the pregnant gilt by a combination of oral, subcutaneous, intramuscular, and intranasal inoculation. Likely, this is a more intense exposure to the APPV, as compared to a natural infection. Thus, it can be hypothesized that neurotropic APPV may induce several signs derived from interference in the CNS, such as an inability to control the extremities if the infection is excessive. However, natural infections with APPV have only been associated with signs of congenital tremor [6, 29, 51, 52].

The results from this study have increased the understanding of the local immune mechanisms in the APPV-infected brain and added to the knowledge of the ultrastructural changes in the brain of piglets with signs of congenital tremor. It also lays the groundwork for a further understanding of the CNS virome in sick and healthy piglets. Several questions, for example how the APPV-infection resolve and how permanent damage to the brain is prevented, still remain to be answered and further research on the topic is needed.

## Conclusion

The aim of the present study was to provide a more comprehensive description of natural cases of congenital tremor type A-II and splay leg, respectively. An APPV-associated cytokine profile, characterized by an upregulation of the gene encoding STING has been identified, as well as a lack of histopathological lesions but the presence of ultrastructural changes in the brain of a piglet with congenital tremor. The study could not identify any viral agent in the spinal cord of piglets with signs of splay leg and the study provides no evidence for a common causative agent or pathogenesis of splay leg and congenital tremor.

## Material and method

### Study population

The study included archived material from 36 new-born piglets; brain tissue from eight healthy control animals, spinal cord tissue from 13 piglets with signs of splay leg, and brain tissue from 15 piglets with signs of congenital tremor. The tissue originated from animals sampled in 2017/2018, as described by Stenberg et al. (2020a), and stored at – 80 °C. Brain tissue (gray matter from the cerebrum) was analysed from piglets withs signs of congenital tremor and tissue from the spinal cord (the thoracic part) was analysed from piglets with signs of splay leg. Different tissues were chosen for analysis since congenital tremor is associated with infections and lesions in the brain (Arruda et al., 2016, de Groof et al., 2016, Postel et al., 2016, Stenberg et al., 2020) whereas splay leg is associated with lesions and infections in the spinal cord (Papatsiros, 2012, Jeong et al., 2017, Schumacher et al., 2021).

The general condition of the piglets, sampling, and farm of origin as well as specific Cq-values and a phylogenetic analysis of the virus is described in detail in Stenberg et al*.,* (2020a). The piglets with signs of congenital tremor originated from five farms and six litters. The piglets with signs of splay leg originated from four farms and eight litters. The healthy piglets originated from four farms and seven litters. The healthy piglets and the piglets with signs of splay leg were APPV-genome negative in the brain and spinal cord tissue, respectively, whereas 13 out of 15 piglets with signs of congenital tremor were APPV-genome positive in the brain tissue [6]. The two APPV-genome negative piglets with clinical signs of congenital tremor were littermates, and the only piglets sampled at that specific farm. In addition, two 5-day old piglets were sampled in January 2022, one with signs of congenital tremor, and one healthy control piglet from a litter containing only healthy piglets. Both piglets originated from a farm experiencing an outbreak of congenital tremor and were only included in the histological and transmission electron microscopy (TEM) analyses. All animal studies were approved by the ethical committee for animal experimentation of Uppsala 2017–02-10 (Dnr 5.8.10–00,431/2017) in accordance with the current European (directive 86/609/EEC) and the Swedish legislation (Djurskyddslag 2018:1192). All methods are reported in accordance with ARRIVE guidelines.

All piglets were transported to and euthanized at the pathological facility at the Swedish University of Agricultural Sciences in Uppsala. The piglets were sedated with an intramuscular injection of tiletamine and zolazepam (Zoletil®, Virbac, Carros, France) and euthanized by an intraperitoneal injection of pentobarbital (Allfatal vet. Apotek Produktion & Laboratorier AB, Malmö, Sweden). To ensure quick and correct handling of the piglets and to minimize the time from death to necropsy to a few minutes the piglets were sedated and euthanized one at a time.

### High-throughput sequencing

#### RNA-extraction

The brain and the spinal cord samples were cryolyzed in ice-cold PBS using a Precellys tissue homogenizer (Bertin Corp. Rockville, MD, USA) and centrifuged for 10 min at 4 000 RCF in 4 °C. The supernatants were transferred to a spin filtrate column (0.45 µm) and centrifuged for 4 min at 4 000 RCF in 4 °C. The filtrates were treated with 2U of TURBO™ DNase (Invitrogen, Thermo Fisher Scientific, Waltham, MA, USA) and DNase I (Invitrogen, Life Technologies, Carlsbad, CA, USA) and incubated for 30 min at 37 °C. Thereafter, a total volume of 200 µL was treated with 5 µL RNase Cocktail™ (Invitrogen, Thermo Fisher Scientific, Waltham, MA, USA) and incubated for 5 min at room temperature.

RNA was extracted using a trizolphenol-chloroform protocol and cleaned using the GeneJET RNA kit (ThermoFisher Scientific, Waltham, MA, USA). During the RNA extraction, an “on-column” DNase digestion was performed using the RNase-Free DNase Set (QIAGEN GmbH, Hilden, Germany). The concentration of the purified RNA was quantified on a Qubit® 2.0 Fluorometer using the Qubit RNA HS assay kit (Thermo Fisher Scientific, Paisley, UK).

#### Library preparation and sequencing

**Fig. 4:**
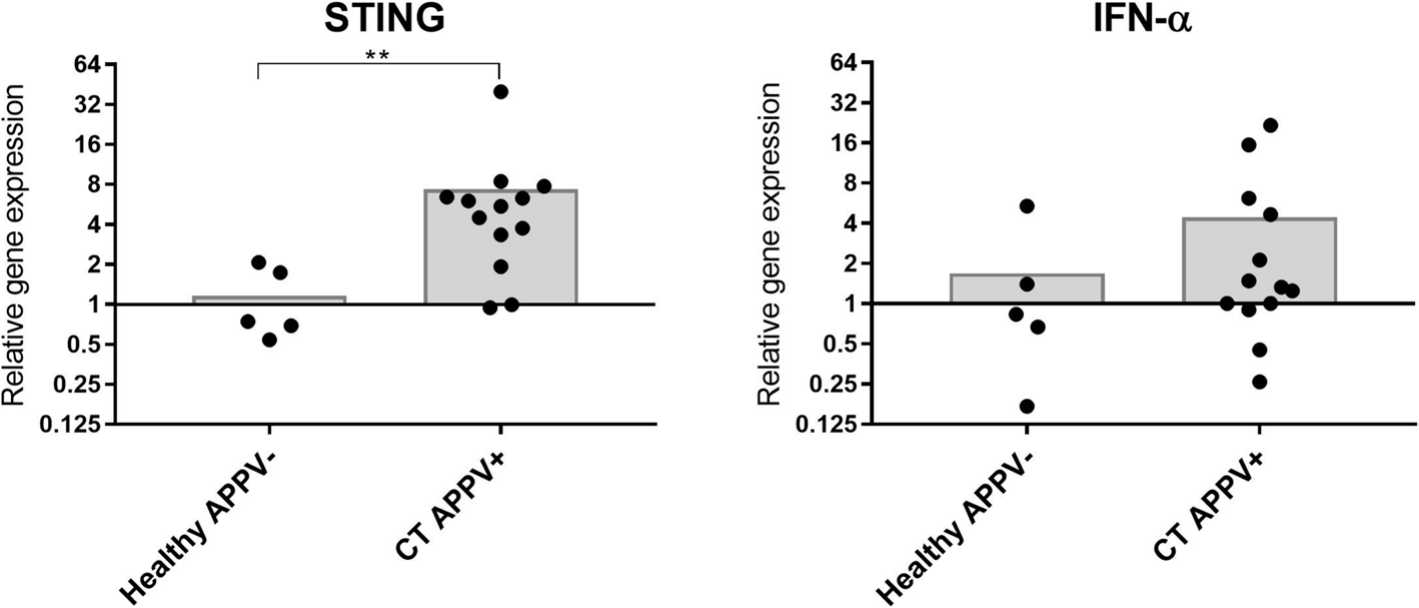
Graph presenting the expression of STING and of IFN-α encoding genes. Significant upregulation, *p* < 0.05, of STING encoding gene in piglets PCR-positive for APPV and with signs of congenital tremor compared to the healthy control piglets. An up-regulation of IFN-α is indicated, however not statistically significant

Sequencing libraries were prepared in two batches, first for sequencing on a MiSeq instrument (Illumina, San Diego, CA, USA) at the National Veterinary Institute, Uppsala, Sweden, and later for sequencing on a NovaSeq 6000 system at the SNP&SEQ Technology Platform at SciLifeLab in Uppsala (Fig. 4).

#### MiSeq sequencing

In the MiSeq sequencing, the eight healthy piglets and six piglets with signs of splay leg were included. Sequencing libraries for the MiSeq run were prepared using the Trio RNA-Seq Library Preparation Kit (NuGEN Technologies, San Carlos, CA, USA). The library concentration was measured using a Qubit® 2.0 Fluorometer (Thermo Fisher Scientific, Paisley, UK) and an Agilent High Sensitivity DNA Kit (2100 Bioanalyzer, Agilent Technologies, Palo Alto, CA, USA). Based on the concentration, pooling and normalization to 2 nM were performed. The library pools were diluted in RNase-free water. Paired-end sequencing was performed with a MiSeq Reagent Kit v3 600 cycles on the MiSeq instrument (Illumina, San Diego, CA, USA) at the National Veterinary Institute, Uppsala, Sweden. The quality of the dataset was assessed using the FastQC software [53].

#### NovaSeq sequencing

In the NovaSeq sequencing, seven piglets with signs of congenital tremor, one from each sampled litter and the two APPV-genome negative piglets as well as one piglet with signs of splay leg were included. The sequencing libraries for NovaSeq were prepared using the Trio RNA-Seq Library Preparation Kit with the Custom AnyDeplete for targeted pig genome depletion (NuGEN Technologies, San Carlos, CA, USA). Library pooling and normalization of 5 ng DNA/µL were performed based on the concentration recorded by Qubit® 2.0 Fluorometer (Thermo Fisher Scientific, Paisley, UK) and TapeStation (Agilent, Santa Clara, CA, USA). RNase-free water was used for the library dilution.

Using the NovaSeq 6000 system, a paired-end 150 bp read length sequencing was performed using an SP flow cell and the v1 sequencing chemistry (Illumina, San Diego, CA, USA). A 1% spike-in with a sequencing library for the phage PhiX was included in the run. The FastQC software [53] was used to assess the quality of the produced dataset.

#### Assembly of sequence reads

To analyse the produced datasets a NextFlow pipeline was implemented. The pipeline included the following steps: quality control and trimming of the reads using FASTP version 0.19.5 [54], removal of the host reads by mapping on *Sus scrofa* genome using bowtie2 version 2.3.5.1 [55], and de novo assembly of the remaining reads using MEGAHIT version 1.2.9 [56]. Taxonomic classification of the reads was performed using Kraken 2 version 2.0.8-beta [57] against the nucleotide non-redundant (*nt*) NCBI database. For the taxonomic classification of the assembled contigs, two methods were used and compared: Kraken2 and DIAMOND version 0.9.24.125 [58]. Kraken2 was run against both the non-redundant protein database (*nr*) and the nucleotide non-redundant (*nt*) NCBI database, and DIAMOND was run against the *nr* protein database. The pipeline is fully available online at GitHub (https://github.com/jhayer/nf-metavir).

The resulting reports were visualised using Pavian [59]. Additionally, specific alignments against the APPV genome (NC_030653.1 Atypical porcine pestivirus 1 isolate Bavaria S5/9 polyprotein gene, complete cds) were performed using Bowtie2 version 2.3.5.1 [55].

### Cytokine analysis

#### RNA isolation and cDNA synthesis

Isolation of RNA from brain tissue and spinal cord was performed as previously described by Stenberg et al. (2020a). The quantity and purity of the extracted RNA were measured by spectrophotometry (NanoDrop ND-1000, NanoDrop Technologies, Montchanin, DE) and Agilent High Sensitivity DNA Kit (2100 Bioanalyzer, Agilent Technologies, Palo Alto, CA, USA). Three of the eight healthy control piglets were excluded due to non-sufficient RNA concentration for the cDNA protocol. Synthesis of cDNA was performed using an input of 1.2 μg RNA per reaction (GoScript Reverse transcription system, Promega). To remove potential contamination of genomic DNA, the RNA was treated with RQ1 RNAse-free DNAse (Promega, Madison, WI, USA), and a -RT control was run parallel to the RNA samples. All samples were diluted 1:5 in RNase-free water and stored at − 20 °C until use.

#### qPCR analysis

Expression of the genes encoding CXCL8, IFN-α, IFN-β, IFN-γ, IFITM3, IL-1β, IL-6, IL-10, and STING was estimated using previously published primer pairs and assay conditions [60]. All samples were run in duplicate reactions of 25 µL with 2 µL cDNA in 23 µL Quantitect SYBR Green PCR mix (Qiagen) on a CFX96 Touch PCR machine (Bio-Rad). The cycling protocol was initiated by a cycle of 15 min at 95 °C followed by 40 cycles of 15 s at 95 °C, 30 s at the assay-specific annealing temperature, 30 s at 72 °C, ending with a melt curve analysis to verify the PCR product. To enable relative quantifications, primer pairs for five reference genes; GAPDH, HPRT, PPIA, RPL32, and YWHAZ were tested for their expression stability in a representative selection of porcine brain tissue using previously established assay conditions [60, 61]. Based on geNorm analysis (qBase^PLUS^, Biogazelle), the genes for GAPDH (M = 1.32; CV = 0.76), HPRT (M = 1.17; CV = 0.42), and RPL32 (M = 1.06; CV = 0.32) were selected for normalization of data. For each cytokine gene, the Cq value was normalized to the geometric mean of the three reference genes. The relative quantity of each gene was calibrated to the mean of the healthy control pigs (*n* = 5). Genes with fold change values < 0.5 or > 2 were regarded as down- or up-regulated.

### Statistical analysis

Statistical analysis was performed using the software Prism 7.0 (Graph-Pad). Differences in the expression of cytokine genes between the APPV negative healthy piglets and APPV positive piglets were calculated using an unpaired t-test on ΔΔCq values. To test for correlation in the expression of STING and IFN-α, a bivariate fit analysis was performed using the JMP® Pro 15.2.0. The model included the observations of the expression of STING and IFN-α from the thirteen APPV-genome positive piglets with signs of congenital tremor. For all tests, p-values below 0.05 were regarded as significant. When indicated, variability of gene expression data is reported as mean FC ± SD.

### Pathology

At necropsy, the brain and spinal cord were immediately removed first to ensure a quick fixation, thereby reducing the risk for post mortem artifacts. The tissue samples were immediately fixed in 10% neutral buffered formalin. Eight sections were taken from the CNS, five from the brain and three from the spinal cord; globus pallidus, the parietal cortex, hippocampus, thalamus, mesencephalon, cerebellum, obex, post colliculus, and the cervical, thoracic-, and lumbar part of the spinal cord. All paraffin-embedded tissue was sectioned at 4 µm and mounted on slides that were routinely stained with haematoxylin and eosin for histologic review. In addition, the cerebellum and spinal cord from three piglets with the highest viral load and one control piglet were stained using luxol fast blue stain and cresyl violet stain.

### Transmission electron microscopy

Two piglets, one with clinical signs of congenital tremor and one healthy control piglet, sampled in 2022, were selected for TEM. Tissue from the cerebellum were cut into cubes of 1 mm^3^ before fixation.

### Fixation and embedding

For transmission electron microscopy, the samples were fixed in 2.5% glutaraldehyde (Ted Pella INC, Redding, CA, USA) + 1% paraformaldehyde (Merck, Darmstadt, Germany) in PIPES (Merck, Darmstadt, Germany) pH 7.4, and stored at 4 °C until further processed. Samples were rinsed with 0.1 M phosphate buffer for 10 min prior to 1 h incubation in 1% osmium tetroxide (TAAB, Aldermaston, England) in 0.1 M phosphate buffer. After rinsing in 0.1 M phosphate buffer, samples were dehydrated by incubation in increasing concentrations of ethanol (50%, 70%, 95% and 99.9%) for 10 min each, followed by 5 min incubation in propylene oxide (TAAB, Aldermaston, England). The tissue samples were thereafter placed in a mixture of Epon Resin (Ted Pella INC, Redding, CA, USA) and propylene oxide (1:1) for 1 h, followed by 100% resin, and left over night. Subsequently, the samples were embedded in capsules in newly prepared Epon resin, left for 1–2 h, and then polymerized at 60 °C for 48 h.

### Sectioning and contrasting

The specimens were cut into semi-thin Sects. (1–2 microns), stained in Toluidine Blue and examined by light microscopy. The blocks were trimmed and ultrathin Sects. (60- 70 nm) were cut in an EM UC7 Ultramicrotome (Leica, Stockholm, Sweden), and then placed on a grid. The sections were subsequently contrasted with 5% uranyl acetate and Reynold’s lead citrate and visualized with Tecnai™ G2 Spirit BioTwin transmission electron microscope (Thermo Fisher/FEI, Hillsboro, Oregon, USA) at 80 kV with an ORIUS SC200 CCD camera and Gatan Digital Micrograph software (both from Gatan Inc, Pleasanton, CA, USA).

## Supplementary Information

**Additional file 1.** 

## Data Availability

The datasets generated and/or analysed during the current study are available in the PRJEB50949 repository at the European Nucleotide Archive. [https://www.ebi.ac.uk/ena/browser/view/PRJEB50949?show=reads, accession number: SAMEA13443979—SAMEA13444000].
