# Supplementary material for: Congenital tremor and splay leg in piglets – insights into the virome, local cytokine response, and histology
Source: BMC Vet Res. 2022 Sep 16;18:348. doi: 10.1186/s12917-022-03443-w (PMC9479355; doi:10.1186/s12917-022-03443-w)
Supplement: Supplementary file 1 — Additional file 1. [file 12917_2022_3443_MOESM1_ESM.docx]

| **Sample id** | **Host** | **Number of reads before QC** | **Read length before QC** | **Number of good quality reads** | **Read length good quality reads** | **% Host mapped** | **Total nr of contigs** | **Viral contigs** |
| --- | --- | --- | --- | --- | --- | --- | --- | --- |
| SL-2406 Sample 8 | Sus scrofa  Spinal cord | 82.193574 M | 151bp, 151bp | 78.102734 M | 108bp, 108bp | 80,2% | 3048 | 1 |
| SL-2406 Sample 9 | Sus scrofa  Spinal cord | 1.048130 M | 151bp, 151bp | 942,672000 K | 118bp, 117bp | 81,72% | 117 | 0 |
| SL-2406 Sample 10 | Sus scrofa  Spinal cord | 38.629224 M | 151bp, 151bp | 37.157866 M | 111bp, 111bp | 80,21% | 3987 | 3 |
| SL-2406 Sample 11 | Sus scrofa  Spinal cord | 32.478690 M | 151bp, 151bp | 30.808594 M | 121bp, 121bp | 79,05% | 8920 | 15 |
| SL-2406 Sample 12 | Sus scrofa  Spinal cord | 5.253810 M | 151bp, 151bp | 4.979272 M | 117bp, 117bp | 79,75% | 1007 | 2 |
| SL-2406 Sample 13 | Sus scrofa  Spinal cord | 1.680402 M | 151bp, 151bp | 1.636422 M | 130bp, 130bp | 14,47% | 13223 | 17 |
| TA-2420 Sample 14 | Sus scrofa  Brain | 19.407866 M | 151bp, 151bp | 19.050090 M | 105bp, 105bp | 89,17% | 5583 | 13 |
| TA-2420 Sample 15 | Sus scrofa  Brain | 26.186510 M | 151bp, 151bp | 25.851982 M | 109bp, 109bp | 89,6% | 9112 | 17 |
| TA-2420 Sample 16 | Sus scrofa  Brain | 29.866836 M | 151bp, 151bp | 29.001994 M | 121bp, 121bp | 87,63% | 15402 | 25 |
| TA-2420 Sample 17 | Sus scrofa  Brain | 52.000906 M | 151bp, 151bp | 50.641918 M | 111bp, 111bp | 88,58% | 12407 | 19 |
| TA-2420 Sample 18 | Sus scrofa  Brain | 788.804000 K | 151bp, 151bp | 758.766000 K | 124bp, 124bp | 89,19% | 183 | 0 |
| TA-2420 Sample 19 | Sus scrofa  Brain | 14.527666 M | 151bp, 151bp | 13.911688 M | 123bp, 123bp | 87,77% | 10090 | 20 |
| TA-2420 Sample 20 | Sus scrofa  Brain | 10.437646 M | 151bp, 151bp | 10.111414 M | 129bp, 129bp | 88,71% | 4605 | 12 |
| TA-2420 Sample 21 | Sus scrofa  Brain | 8.382254 M | 151bp, 151bp | 8.132066 M | 132bp, 132bp | 87,93% | 6148 | 16 |
| TC-2516-Sample30 | Sus scrofa Brain | 75.353234 M | 151bp, 151bp | 72.665870 M | 131bp, 131bp | 78,32% | 107083 | 104 |
| TC-2516-Sample31 | Sus scrofa Brain | 8.325108 M | 151bp, 151bp | 8.084110 M | 127bp, 127bp | 78,95% | 16949 | 15 |
| TC-2516-Sample32 | Sus scrofa Brain | 22.087286 M | 151bp, 151bp | 21.406802 M | 127bp, 127bp | 82,13% | 41137 | 18 |
| TC-2516-Sample33 | Sus scrofa Brain | 11.704902 M | 151bp, 151bp | 11.337184 M | 130bp, 130bp | 80,80% | 21428 | 1 |
| TC-2516-Sample34 | Sus scrofa Brain | 8.516168 M | 151bp, 151bp | 8.216892 M | 126bp, 126bp | 82,10% | 18109 | 2 |
| TC-2516-Sample35 | Sus scrofa Brain | 6.294360 M | 151bp, 151bp | 6.122686 M | 130bp, 130bp | 84,23% | 11957 | 0 |
| TC-2516-Sample36 | Sus scrofa Brain | 5.911670 M | 151bp, 151bp | 5.711310 M | 125bp, 125bp | 84,34% | 10444 | 2 |
| TC-2516-Sample37 | Sus scrofa Spinal cord | 2.296270 M | 151bp, 151bp | 2.074764 M | 121bp, 121bp | 79,23% | 143 | 0 |
